# Imaging the Distribution of Sodium Dodecyl Sulfate in Skin by Confocal Raman and Infrared Microspectroscopy

**DOI:** 10.1007/s11095-012-0748-y

**Published:** 2012-04-04

**Authors:** G. Mao, C. R. Flach, R. Mendelsohn, R. M. Walters

**Affiliations:** 1 Johnson & Johnson Consumer Companies, Inc., 199 Grandview Rd., Skillman, New Jersey 08558-9418 USA; 2 Department of Chemistry, Rutgers University, Newark, New Jersey USA

**Keywords:** confocal Raman microscopy, Infrared (IR) imaging, permeation/penetration, skin, surfactant

## Abstract

**Purpose:**

To image SDS distribution across different skin regions, to compare the permeability difference between porcine and human skin, and to evaluate the interaction between SDS and skin.

**Methods:**

Full thickness porcine and human skin was treated with acyl chain perdeuterated SDS (SDS-d_25_) at room temperature and at 34 °C for 3, 24 and 40 h. SDS distribution in skin was monitored by confocal Raman and IR microspectroscopic imaging. Permeation profiles of SDS-d_25_ in skin were derived from the band intensities of the CD_2_ stretching vibrations. The interaction between SDS and skin was monitored through the CH_2_ and CD_2_ stretching frequencies and the Amide I and II spectral region.

**Results:**

SDS-d_25_ penetrates both porcine and human skin in a time and temperature-dependent manner, with slightly higher permeability through the stratum corneum (SC) in porcine skin. When SDS permeates into the SC, its chains are more ordered compared to SDS micelles. The secondary structure of keratin in the SC is not affected by SDS-d_25_.

**Conclusion:**

The spatial distribution of SDS-d_25_ in skin was obtained for the first time. Infrared microscopic imaging provides unique opportunities to measure concentration profiles of exogenous materials in skin and offers insights to interaction between permeants and skin.

**Electronic supplementary material:**

The online version of this article (doi:10.1007/s11095-012-0748-y) contains supplementary material, which is available to authorized users.

## INTRODUCTION

Stratum corneum (SC), the topmost layer of the epidermis, provides a vital barrier function in intact skin. SC structure, often portrayed as a brick and mortar assembly, is composed of anucleated corneocytes embedded in a continuous highly ordered multi-lamellar, lipid matrix (1). The SC lipid matrix provides the main barrier to water loss and permeation of exogenous substances. A primary function of the underlying viable epidermis (VE) is to generate the SC as its outer layers are continuously shed. Beneath the epidermis lies the dermis (Der), a dense, supportive fibroelastic connective tissue with extensive vascular and nerve networks along with excretory and secretory glands.

The lateral packing and lamellar phases of the highly ordered SC lipid matrix have been well studied by various biophysical techniques. In both human and porcine SC, two lamellar phases have been identified by X-ray diffraction (2,3) whereas the nature of the lateral lipid packing, as studied by X-ray and FTIR (4–7) is shown to vary between species. SC lipids are predominantly packed in ordered orthorhombic and hexagonal phases with some (small amounts) disordered lipid also likely present. Keeping in mind sample to sample variation, which is noteworthy, the balance of orthorhombic to hexagonal phase appears to be much greater in human compared to porcine SC. Compositional differences in ceramide classes and fatty acid chain lengths have also been delineated (8,9) between human and porcine SC lipids. A range of temperatures (30–40 °C) have been reported for the orthorhombic to hexagonal phase transition in human SC while the transition has been difficult to measure in porcine SC. Overall at skin surface temperature (~34 °C), a fair amount of orthorhombic phase remains in human with lesser amounts in pig SC.

Surfactants, one of the most widely used components of personal care products and also studied as excipients for drug delivery systems, are recognized as skin irritants. Sodium dodecyl sulfate (SDS) has been extensively studied as a model surfactant in skin research (10,11). Increases in transepidermal water loss (TEWL) following SDS treatment have been reported (12–16). In addition to damaging the skin barrier, SDS permeation causes irritation and inflammation (13,17). It also alters barrier renewing processes by affecting keratinocyte differentiation (18,19) and desquamation (18). SDS evidently not only interacts with the SC but also with deeper skin layers. Thus, its permeation and distribution in skin is of substantial interest.

To date, SDS permeation has been evaluated with a variety of techniques including diffusion cells (20) and patch testing (12,13) with analytical determination of SDS concentration via GC/LC-MS or radioactivity measurements (20). In addition, recent studies of isolated human SC from our laboratories (21) have analyzed the effects of SDS on the conformational order, packing, and phase behavior of the endogenous lipids. However, detailed information concerning the spatial distribution of surfactants in different skin regions is lacking.

Over the past 15 years, confocal Raman microscopy and IR microspectroscopic imaging have evolved as convenient approaches to evaluate permeation of topically applied agents in skin (22–26). The penetration of exogenous components is tracked from the spatial distribution of their characteristic vibrational bands. Agents with isotopically labeled functional groups increase the specificity of the method without affecting permeation. In addition to tracking the permeation of exogenous materials through skin, Raman and IR microspectroscopic imaging can monitor molecular structure changes (e.g. lipid chain ordering and protein secondary structure) induced in skin constituents by the exogenous agents. In the current study, we utilize IR imaging in a novel way, namely, to quantitatively report on the spatial distribution of the concentration of SDS in the SC, VE, and dermis and to evaluate the interaction between SDS and skin components at the molecular level. To the best of our knowledge, this is the first reported quantitative application of IR imaging.

## MATERIALS AND METHODS

### Chemicals

Chain perdeuterated sodium dodecyl sulfate (SDS-d_25_), 98 atom% D, was purchased from Sigma-Aldrich (St. Louis, MO).

Skin biopsies from Yucatan white, hairless pigs in the vicinity of pig ribs were purchased from Sinclair Research Center Inc. (Columbia, MO). Human abdominal skin (otherwise to be discarded) from plastic surgery was obtained from dermatological offices, with informed consent and approval of the institutional ethics committee. Skin specimens were fast frozen in liquid nitrogen after removal of subcutaneous fat tissue and stored at −20 °C for no longer than 1 year prior to use.

### Sample Preparation

SDS-d_25_ was dissolved at about nine times its critical micelle concentration (CMC) in 1× PBS (12.5 mg/mL or 40.8 mM), a concentration similar to typical dermal exposure to personal care cleansers after dilution. Skin samples were cut to ~10 × 10 mm^2^ surface area with a thickness of 2.5 mm and thawed at room temperature. Sample surfaces were cleaned with wet cotton swabs and excess moisture was removed. For SDS treatment, skin samples were secured to home-built diffusion type cells with the SC facing the donor chamber. The surface area of the donor chamber is ~50 mm^2^ with a diameter of 8 mm. ~120 μL of the SDS-d_25_ solution was added to the donor chamber while the receptor chamber was left empty. Samples, wrapped with parafilm to minimize solvent evaporation and to keep skin hydrated, were incubated at room temperature or at 34 °C for 3, 24 or 40 h. Excess SDS-d_25_ was removed after incubation and the skin surface was cleaned with wet cotton swabs.

Duplicate porcine skin samples were treated under various conditions for Raman measurements. After treatment, skin samples were transferred to a brass sample holder and kept enclosed in the holder with a glass cover slide for confocal Raman measurements. Both porcine and human skin was used for IR imaging studies and duplicate skin samples were prepared for the bulk of the IR measurements. Skin sections, ~7 μm thick, were microtomed perpendicular to the skin surface for IR imaging. Eight to twelve microtomed slices were collected from each skin preparation, four of which were analyzed with IR imaging.

### Confocal Raman Microscopy/IR Microspectroscopic Imaging

Raman spectra were acquired with a Raman microprobe (Kaiser Optical Systems, Inc., Ann Arbor, MI). The instrument has been previously described (27). Excitation is achieved with a 785 nm solid state diode laser. Approximately 7 mW of single mode power is focused with a 100× oil immersion objective to a volume of ~2 μm^3^ within the sample. Spectra were acquired using a 60-second exposure time, three accumulations, and cosmic ray correction. Confocal maps were obtained at room temperature from 10 lines of spectra collected perpendicular to the skin surface with a step size of 5 μm.

IR images of skin sections were collected with a Perkin-Elmer Spotlight 300 system (PerkinElmer Life and Analytical Sciences, Inc., Waltham, MA) utilizing the transmission mode with a 6.25 μm pixel size. 32 scans with a spectral resolution of 4 cm^−1^ were averaged for each pixel. The IR image size generated from each skin sample was 300 × 200 μm, with 48 pixels in the direction perpendicular to the skin surface and 32 pixels in the direction parallel to the skin surface. Visible images of the sampled skin regions were acquired with the microscope integrated into the Spotlight 300 system.

We note that the methylene scissoring and rocking modes, which are uniquely sensitive to acyl chain packing phases, are not discussed in the current set of experiments. The rocking modes (715–735 cm^−1^ region) are out of range for the imaging detector and the scissoring bands (1,455–1,480 cm^−1^ region) lack sufficient signal to noise ratios for data analysis in spectra of the ~7 μm thick skin sections.

### Analysis of IR and Raman Data

Visible microscopic images of skin samples are presented as acquired without additional processing. Vibrational microspectroscopic images were created from Raman and IR spectral data with ISys 3.1 software (Malvern Instruments, UK). Spectra extracted from the images were analyzed with Grams/32 (Galactic Industries Corp., Salem, NH). Figures were prepared for presentation with SigmaPlot 2000 (SPSS Inc., Chicago, IL).

The factor analysis algorithm in ISys 3.1 software was utilized in the current work to differentiate and classify skin regions based on the 2,830–3,000 cm^−1^ spectral region. Factor analysis requires for its implementation the prior availability of PCA scores and loadings. The latter are acquired by unfolding the image cube into a two dimensional array, **X**, with pixel spectra along each row. PCA requires decomposition of the data matrix **X** into **Σ**_j_ **S**_**j**_ **L**_**j**_. Each **L**_**j**_ is a 1 x k loading vector, where k is the spectral dimension of the data matrix. Each **S**_**j**_ is a score vector. The product **S**_**j**_ **L**_**j**_ is the fractional variance of **X** accounted for by the j’th principal component.

The loading vectors resulting from the PCA calculation are not pure component spectra. Factor analysis seeks transformations of the PCA loading vectors to the true underlying (chemical) factors. In the current case, each factor is the sum of spectra from the chemical constituents of the tissue, which contributes to the variance at a particular site. A set of scores, **S**, is generated by S = X*L’, where **L’** is the transpose of the matrix of normalized loadings.

Factor analysis is carried out using a score segregation routine. The analysis seeks to detect simple patterns in the relationships between observed variables in order to reduce the dimensionality of the data. Score segregation begins by normalizing PCA scores which may then be sharpened by raising them to a power specified by an acceleration parameter. Factor loadings are calculated according to ${\left( {{\mathbf{S}}\prime {\mathbf{S}}} \right)^{ - {1}}} * {\mathbf{S}}\prime * {\mathbf{X}}$. Usually 3–6 significant factors are observed.

### Determination of SDS-d_25_ Concentration in IR Images

SDS chain perdeuteration shifts the methylene stretching frequencies to a spectral region free of interference from endogenous skin vibrations. As IR spectra of skin samples were acquired in the transmission mode, Beer’s law is applicable. Molar extinction coefficients were derived from the peak area of the symmetric CD_2_ stretching mode (ν_sym_CD_2_) for SDS-d_25_ in both aqueous and ethanolic solutions using a fixed path length (15 μm) CaF_2_ cell. SDS-d_25_ was not sufficiently soluble in any additional solvents of greater hydrophobicity to acquire IR spectra of adequate quality. Measured values were 3.62 × 10^3^ mol^−1^dm^3^cm^−1^ for SDS-d_25_ in water and 4.11 × 10^3^ mol^−1^dm^3^cm^−1^ in ethanol. Correlation coefficients from the Beer’s plots were 98.6 % and 98.9 % for aqueous and ethanol solutions respectively, which demonstrates the very high precision of using ν_sym_CD_2_ peak area to calculate SDS-d_25_ concentration. We assume a mean extinction coefficient of 3.87 × 10^3^ mol^−1^dm^3^cm^−1^ to calculate SDS-d_25_ concentration in skin.

We are well aware that changes in SDS-d_25_ structure/environment may substantially alter the extinction coefficients from those determined in ethanol or water. Chain methylene stretching vibrations are sensitive monitors of conformational order, which in turn defines elements of the micellar structure and correlates to IR band intensity/extinction coefficients. Comparison of SDS-d_25_ ν_sym_CD_2_ stretching frequencies in various skin regions to those in standard solutions provides an estimation of the conservation of the extinction coefficients in skin.

The conformational order of the chains in a 40.8 mM SDS micellar solution is characterized by an observed υ_sym_CD_2_ of 2095.1 cm^−1^ which is quite similar to that of SDS in the dermis (2094.4 cm^−1^ with calculated average concentration of 36.9 mM). This similarity in IR frequencies and concentrations indicates that the chain conformation in solution and in the dermis are similar, and further suggests that the extinction coefficients obtained for micelles can be transferred to SDS in the dermis with reasonable accuracy (5–10 %). In contrast, the average υ_sym_CD_2_ of SDS chains is 2091.6 cm^−1^ and 2092.3 cm^−1^ for SDS in SC and VE respectively, where conformational order has increased compared to the SDS in the standard solutions (2095.1 cm^−1^ as stated above). SDS chain ordering occurs on cooling below the Krafft point causing a frequency shift in υ_sym_CD_2_ to 2089.4 cm^−1^ along with a ~38 % increase in peak area and therefore in extinction coefficient. Thus, the calculated concentration of SDS in the SC and VE using the average extinction coefficient (described above) is likely to be higher than the true value. Since the IR frequency shift is not a linear function of the extent of conformational order, the uncertainty caused by chain ordering in the SC and viable epidermis is difficult to ascertain. The calculated concentration is expected to be an overestimation by a maximum of 38 % but is more likely to be ~20 %.

## RESULTS

**Fig. 1:**
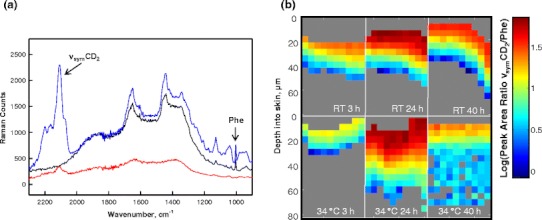
(**a**) Raman spectra (900–2,300 cm^−1^ region) of a 12.5 mg/ml SDS-d_25_ solution (*red*), and the SC region of porcine skin before (*black*) and after (*blue*) SDS-d_25_ treatment. The ν_sym_CD_2_ from SDS-d_25_ and the phenylalanine ring-breathing mode (Phe) from skin are noted in the spectra. (**b**) Confocal Raman image planes depicting SDS-d_25_ distribution in porcine skin following treatment at room temperature (RT) and 34 °C for 3, 24, and 40 h. The skin surface is presented at the top of each image plane. Pixels with a CD_2_ peak height smaller than 50 Raman counts were indistinguishable from the noise level and are excluded. These pixels are colored in *gray* mostly at the bottom of each image plane. The SDS-d_25_ concentration distribution in skin is displayed as the logarithm of the integrated peak area ratio of ν_sym_CD_2_/Phe. Treatment time and temperatures are noted in the images.

Confocal Raman microscopy provides a convenient approach to track the permeation of exogenous agents in skin without complicated sample preparation. The ν_sym_CD_2_ band between 2,050 and 2,150 cm^−1^, shown in Fig. 1a, is utilized to track the extent of SDS-d_25_ permeation. The ring-breathing mode of phenylalanine (phe) at ~1,004 cm^−1^ arising from skin proteins is used as an internal standard to normalize for Raman intensity loss as a function of depth in the skin. Also shown in Fig. 1a are spectra of untreated porcine skin and an SDS-d_25_ solution.

To evaluate the effects of incubation time and temperature on SDS-d_25_ permeation, porcine skin was treated with SDS-d_25_ at both room temperature and 34 °C, a temperature close to skin surface temperature, for 3, 24 and 40 h. The distribution of SDS-d_25_ is shown in Fig. 1b. The skin surface is presented at the top of each image plane. The SC of porcine skin is typically 15–30 μm thick and 3–6 pixels were collected from SC region. The relative SDS-d_25_ concentration in the skin is plotted as the logarithm of the area ratio of the ν_sym_CD_2_ contour to the 1,004 cm^−1^ phe vibration. The logarithmic presentation permits a wider range of concentrations to be delineated in the color coding scheme of the images. Pixels with a ν_sym_CD_2_ peak height smaller than 50 Raman counts were not distinguishable from the noise level and are excluded. These pixels are colored in gray mostly at the bottom of each image plane. At room temperature, SDS-d_25_ was mostly limited to the top 20–30 μm of skin; the highest SDS-d_25_ intensity was observed for samples treated for 24 h. Detectable levels of SDS-d_25_ permeated much deeper into skin when incubated at 34 °C, especially after 24 and 40 h treatments.

The observation of lower SDS-d_25_ intensity in the top layers of skin following 40 h compared to 24 h treatment was unanticipated. Also, less SDS-d_25_ was present in the top skin layers when treated at 34 °C compared to room temperature for 3 h and 40 h treatment times. Although there is significantly less orthorhombic phase in porcine compared to human SC (see Introduction), these observations may result from a small temperature-dependent decrease in the ratio of orthorhombic to hexagonally packed lipids. We also suspect that SDS-d_25_ disrupts the SC lipid structural integrity. Both perturbations appear to modify SDS solubility in the SC.

While Raman microscopy is convenient for the study of SDS-d_25_ permeation into the SC, the diminution of Raman intensity with depth precludes the possibility of obtaining high quality spectral data at depths greater than ~85 μm. Therefore, IR imaging was used to track SDS-d_25_ permeation at greater skin depths.

**Fig. 2:**
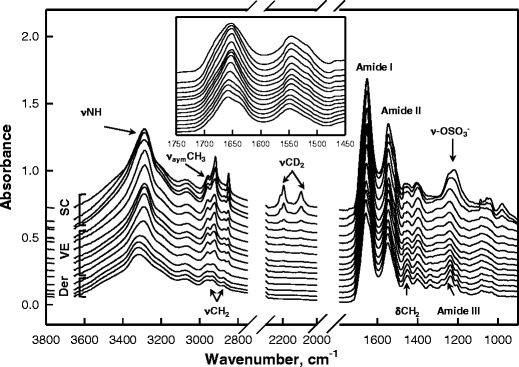
A set of IR spectra from human skin after 24 h SDS-d_25_ treatment at 34 °C. Spectra from the skin surface are shown on top while spectra from deeper skin regions are at the bottom, with a 6.25 μm depth increment between traces. Skin regions, from which the spectra are shown, are labeled and relevant spectral assignments are noted. The same spectra are expanded upon in the Amide I and II region in the inset.

Figure 2 shows a set of IR spectra from human skin samples treated with SDS-d_25_ for 24 h at 34 °C. Spectra are overlaid and labeled according to their position in skin. The step size between each collected spectrum is 6.25 μm. The CH_2_ asymmetric and symmetric stretching modes (ν_asym_CH_2_ and ν_sym_CH_2_, respectively) at ~2,920 cm^−1^ and ~2,850 cm^−1^ and the CH_2_ scissoring mode (δCH_2_) contour between ~1,460–1,475 cm^−1^ arise mostly from skin lipids. These frequencies are sensitive to lipid acyl chain conformation and packing order (28). The N-H stretching mode (νNH) at ~3,290 cm^−1^, CH_3_ asymmetric stretching mode, (ν_asym_CH_3_) at ~2,950 cm^−1^ and the Amide I and Amide II bands (better shown in Fig. 2 inset) at ~1,650 cm^−1^ and ~1,550 cm^−1^ arise mostly from vibrations of skin proteins. Features arising from SDS-d_25_ include the CD_2_ acyl chain stretching modes (~2,050–2,250 cm^−1^) and the alkyl sulfonate stretching bands at ~1,210 cm^−1^. The latter overlap with the protein Amide III modes from skin and are therefore not analyzed further.

**Fig. 3:**
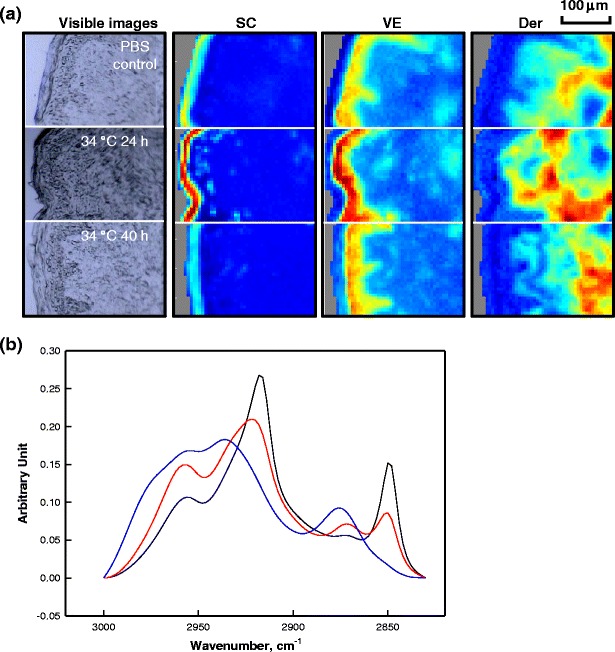
(**a**) Microscopic images and factor analysis score images (color coding of scores: *red*>*yellow*>*blue*) depicting different regions of human skin from a PBS control experiment (24 h treatment at 34 °C) and following SDS-d_25_ treatment for 24 and 40 h at 34 °C. The skin surface is located at the left of each image plane. Factor analysis was conducted over the CH stretching region (2,830–3,000 cm^−1^). (**b**) Factor loadings map to high scores in the following regions of human skin: SC (*black*), VE (*red*) and Der (*blue*).

Delineation of skin regions (e.g. SC vs VE vs dermis) is facilitated by the spectral information regarding skin structure and (bio)chemical composition in the IR images. Whereas skin regions can be approximately visualized from the visible microscopic images presented in the first column of Fig. 3a, IR imaging provides a more precise way to differentiate skin regions. As shown in Fig. 2, IR spectra of skin, especially the νCH_2_ and Amide region, change systematically with depth into skin and when moving from one skin region to another. In the current case, factor analysis is applied to the 2,830–3,000 cm^−1^ range of the spectral images to delineate different skin regions. The results for human skin treated with SDS-d_25_ for 24 h or 40 h at 34 °C along with a control sample consisting of skin treated with PBS buffer for 24 h at 34 °C, are shown in Fig. 3. The three factor loadings shown in Fig. 3b clearly differentiate the spectral characteristics of the SC, VE and dermis and are nearly identical to the actual spectra from each skin region shown in Fig. 2. Factor score images are shown for each loading spectrum in columns 2–4 of Fig. 3a. The color coding measures the correlations between the actual spectrum at each pixel and the factor loading, with red showing the highest similarity and blue, the lowest. The thickness of the SC is observed to be about ~20 μm as shown in the second column of Fig. 3a. The thickness of the VE varies between samples and also laterally within each sample due to the papillae undulations in the dermis. The PBS control and SDS-d_25_ treated SC are slightly thicker compared to untreated skin samples (data not shown). Thus, the observed SC swelling presumably results predominantly from PBS rather than from SDS-d_25_ penetration and is not discussed further.

**Fig. 4:**
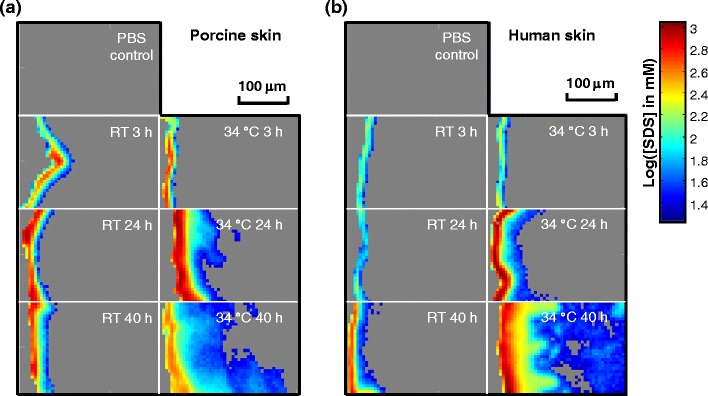
IR image planes showing the SDS-d_25_ concentration distribution in (**a**) porcine and (**b**) human skin following various SDS-d_25_ treatment conditions along with control samples treated with PBS for 24 h. SC regions are shown on the left-hand side of each image. SDS-d_25_ concentrations were calculated from the integrated peak area of ν_sym_CD_2_ and are shown on a logarithm scale. Treatment time and temperatures are noted in the images.

The ν_sym_CD_2_ band intensities in the IR are utilized to track SDS-d_25_ levels in skin. As discussed in the experimental section, the concentration of SDS-d_25_ (imaged in Fig. 4) was determined by Beer’s Law with extinction coefficients estimated from SDS-d_25_ aqueous and ethanolic standard solutions. The approach demonstrates a unique advantage of IR imaging. The results for porcine and human skin are depicted in Fig. 4a and b, respectively, with the skin surface shown on the left-hand side of each image. The SDS-d_25_ spatial distribution in the two species for similar treatment conditions exhibits some differences. For porcine skin shown in Fig. 4a, SDS-d_25_ was mostly confined to the SC regions when samples were treated at RT or incubated for 3 h at 34 °C. For the remaining samples treated at 34 °C, SDS-d_25_ permeated into the VE when treatment time increased to 24 h and diffused into the first 100–200 μm of the dermis following 40 h incubation. Similar or slightly higher SDS-d_25_ concentration in the SC was observed with samples treated at RT compared to 34 °C. The highest SDS-d_25_ concentration in SC was found for the 34 °C 24 h sample. This is consistent with confocal Raman results shown in Fig. 1b. For human skin shown in Fig. 4b, SDS was present only in SC regions for samples with treatment conditions of RT 3 h, RT 24 h and 34 °C 3 h. SDS-d_25_ penetrated into the VE after 40 h treatment at RT or 24 h incubation at 34 °C and continued to diffuse throughout the dermis after 40 h incubation at 34 °C. In contrast to porcine skin, higher SDS-d_25_ concentration in the SC was detected in human skin samples treated at 34 °C compared to RT after the same incubation time. The highest SDS-d_25_ concentration in human SC was observed with skin treated for 24 h and 40 h at 34 °C.

**Fig. 5:**
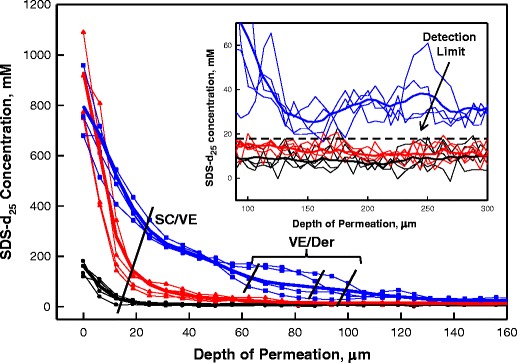
Sample depth profiles of SDS-d_25_ concentrations in human skin from different lateral skin positions treated for 3 (•), 24 (), and 40 h () at 34 °C. The data points from the same lateral position in skin are connected with lines of the same color for better presentation. The bold lines are the average SDS-d_25_ concentration depth profiles for the entire image after each treatment. The inset is the SDS-d_25_ concentration in the deeper region of skin, 100-300 μm beneath the surface. Symbols for each data point are omitted in the inset.

Line profiles depicting SDS-d_25_ concentration with permeation depth were extracted from the images of the human skin 34 °C experiments (Fig. 4b) and are presented in Fig. 5. Four sample lines along with one line averaging laterally across the entire skin image are shown for each incubation period with SDS-d_25_ concentrations at deeper skin sites more clearly shown in the inset of Fig. 5. Interference from the weak water association band between 2,000 and 2,500 cm^−1^ precludes detection of CD_2_ bands with peak heights lower than ~1.9 milliabsorbance units, providing a detection limit of ~17 mM for SDS-d_25_ concentration in skin. The same general time dependencies of SDS-d_25_ permeation are seen in the line profiles of Fig. 5 as noted in the images of Fig. 4b, while SDS-d_25_ concentration progression with depth into skin is more clearly presented with a linear scale. The SDS-d_25_ concentrations decreased rapidly with depth into the SC and became largely unchanged across the VE. Following an additional, less steep concentration gradient for the 40 h sample across the epidermal/dermal boundary (individual line profiles), SDS-d_25_ is distributed more or less evenly throughout the dermis at a concentration of ~32 mM (see Fig. 5 inset). As noted in Fig. 5, the line profiles display a range of positions of the VE/Der boundary due to the undulations of the papillary dermis (Fig. 3). As with most dermal transport studies, the thermodynamics and kinetics of the SDS-d_25_ permeation cannot be deconvoluted in these experiments. The resultant SDS-d_25_ concentration observed in the skin is determined by both the solubility and diffusivity. Thermodynamically, these SDS-d_25_ concentration profiles are presumably influenced by the variation in SDS-d_25_ partition coefficients across different skin regions. Kinetically, the different regions of skin present different barriers to SDS-d_25_ permeation, with the most effective barrier (most rapid concentration decrease) present in the SC, and a possible secondary barrier at the epidermal/dermal junction (discontinuity in concentration profile), while the dermis seems to provide minimal barrier function to SDS-d_25_ permeation. It is noted that the epidermal/dermal junction is conventionally believed to provide a barrier for macromolecules and cell movements while being permeable to small molecules (29).

**Fig. 6:**
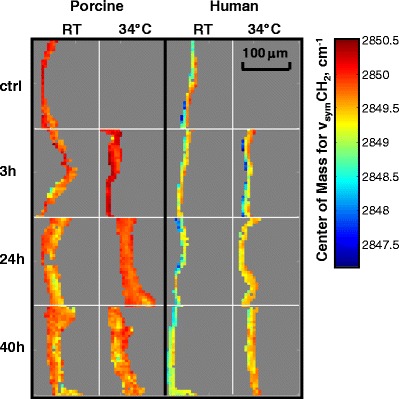
IR image planes depicting intercellular lipid order probed by center of mass frequencies for the ν_sym_CH_2_ in SC regions following various SDS-d_25_ treatment conditions along with an untreated control for porcine skin and a tape stripped (two tape strips) control for human skin. Treatment conditions are noted in the figure. The SC region was delineated by factor analysis as shown in Fig. 3.

Endogenous SC lipid structural properties can be probed by analyzing the CH_2_ acyl chain stretching vibrations in the IR spectra. As mentioned above, the ν_sym_CH_2_ stretching frequency is a sensitive measure of lipid acyl chain conformational order; the lower the frequency, the greater the order. These frequencies are also slightly affected by lipid packing. Images depicting the ν_sym_CH_2_ frequency for porcine and human SC exposed to various SDS-d_25_ treatment conditions together with untreated controls are presented in Fig. 6. For the untreated control samples, the average lipid acyl chain conformational order within the human SC (with sebum removed by tape stripping) is slightly higher with an average ν_sym_CH_2_ frequency of 2849.2 cm^−1^ compared to that of 2849.8 cm^−1^ for porcine SC. Slightly lower ν_sym_CH_2_ frequencies in the SC are also observed for all the SDS-d_25_ treated human compared to porcine samples. It is more difficult to access the effects of SDS-d_25_ treatment temperature and time on SC lipid order. SDS-d_25_ treatment slightly increased ν_sym_CH_2_ frequency for porcine SC only following 3 h incubation at 34 °C compared to the porcine control, whereas human SC treated for 24 h and 40 h at 34 °C display a slight increase in frequency compared to its control sample.

**Fig. 7:**
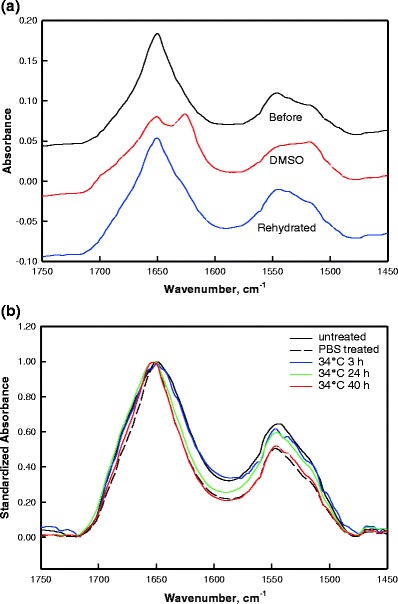
The Amide I and II contours of (**a**) isolated corneocytes before (*black*) and after (*red*) treatment with dimethyl sulfoxide (DMSO) along with the spectrum after rehydration (*blue*); (**b**) average spectra of the SC region of human skin: untreated (*solid black*), PBS treatment for 24 h at 34 °C (dash black), and SDS-d_25_ treatment for 3 (*blue*), 24 (*green*) and 40 h (*red*) at 34 °C. Spectra are normalized to Amide I peak height and baseline corrected over the 1,478–1,722 cm^−1^ region.

To evaluate the effect of SDS-d_25_ on keratin structure, the conformation-sensitive Amide I and II spectral regions were monitored in the SC. As an example, prior studies from the Rutgers laboratory (27) (Fig. 7a) revealed large changes in both the Amide I and II band contours of isolated corneocytes following DMSO treatment, which were consistent with a major α-helix to β-sheet interconversion. Such changes in the Amide spectral region were mostly reversible following corneocyte rehydration. The sensitivity of the Amide spectral region to keratin structure changes was thereby proven.

In contrast to the large perturbation caused by DMSO, SDS-d_25_ causes at most, small changes in the Amide I band contour. Averaged spectra of the Amide I and II region from human SC before and after SDS-d_25_ treatment at 34 °C along with 24 h PBS treatment at 34 °C are shown in Fig. 7b. Similar changes in this spectral region following the same SDS-d_25_ treatment conditions were observed for porcine skin (data not shown). Spectra are baseline corrected between 1,478 and 1,722 cm^−1^ and normalized to the Amide I peak height to account for sample-to-sample variation in protein content. Band narrowing on the low frequency side of the Amide I contour and a decrease of the Amide II/Amide I peak height ratio was observed following PBS treatment for 24 h at 34 °C. No additional perturbation was observed when SDS was present. The origin of these changes is beyond the scope of this manuscript and is currently being investigated.

**Fig. 8:**
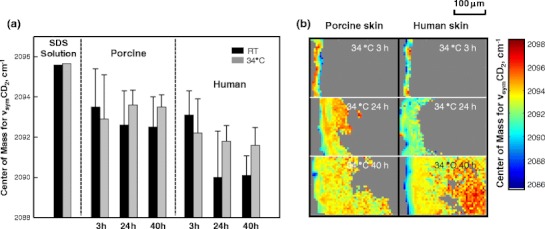
(**a**) IR center of mass frequency for the ν_sym_CD_2_ of SDS-d_25_ in aqueous solution and in the SC region of porcine and human skin treated with SDS-d_25_ for 3, 24, and 40 h at room temperature (*black*) and 34 °C (*gray*). We note that the error bars (standard deviation) do not wholly reflect lack of precision in the measurements but rather predominantly arise from heterogeneity in the skin. (**b**) Image planes showing the center of mass frequency of ν_sym_CD_2_ in both porcine and human skin after treatment for 3, 24 and 40 h at 34 °C. The average ν_sym_CD_2_ frequency for the micellar SDS-d_25_ solution is marked as the *black line* on the scale bar.

Besides their utility as intensity markers for SDS-d_25_ permeation, the CD_2_ stretching frequencies are also valuable for tracking chain conformational order in SDS-d_25_. Figure 8a shows the ν_sym_CD_2_ frequency of an SDS-d_25_ solution at 9 times the CMC and averaged frequencies for SDS-d_25_ that has permeated into porcine and human SC under a variety of treatment conditions. Significantly lower frequencies were observed for the SDS-d_25_ acyl chains when the surfactant was present in the SC compared to its micellar solutions. The intercellular SC lipids clearly have an ordering effect on the permeated SDS-d_25_. The human SC lipids have stronger ordering effects on SDS compared to porcine SC lipids, as shown by the lower ν_sym_CD_2_ frequency. Significantly smaller effects were noted in human skin for the RT 3 h treatment than for longer incubation times, indicating that at room temperature, SDS-d_25_ chain ordering by SC lipids proceeds more slowly than its penetration. SDS-d_25_ acyl chains in the SC were much less ordered when skin was treated at 34 °C for 24 h or 40 h, compared to room temperature for the same incubation times. Since the chain order of SDS-d_25_ is affected by SC lipid structure, along with the temperature effect on lipid packing, these observations tend to indicate a more severe disruption of the endogenous SC lipid acyl chain conformational order and/or packing at higher temperatures caused by the SDS-d_25_ and is in agreement with the slightly higher endogenous ν_sym_CH_2_ frequency for human SC treated at 34 °C shown in Fig. 6. This suggests that ν_sym_CD_2_ frequency from SDS-d_25_ may serve as a sensitive probe of SC lipid order.

Images of SDS-d_25_ acyl chain order in the SC, VE and dermis are shown in Fig. 8b. Compared to the ordering effects of the SC on the SDS-d_25_ chains, the VE and dermis have a smaller effect, presumably due to the lack of highly ordered lipid structure in those skin regions. As noted on the scale bar in Fig. 8b, the ν_sym_CD_2_ frequency of the SDS-d_25_ in dermis is close to that in solution.

## DISCUSSION

A primary focus of skin biology, pharmacology, and biophysics is to develop tracking methods for the permeation and distribution of exogenous agents applied to skin. Franz or similar diffusion cells are typically used to apply exogenous agents to skin. Detection and quantification of the agents following permeation is commonly accomplished via HPLC or radioactivity measurements of extracted material from isolated skin regions (SC, VE and dermis) and of the solution from the receptor chamber. These approaches provide excellent detection sensitivity but very limited spatial resolution. Alternatively, tape stripping can be applied to provide depth dependent information in the SC region, albeit with inherent ambiguity as to the actual depth without an ancillary method to correct the amount of SC removed by each tape strip. The cohesive strength of the SC increases with depth (30) and fewer corneocytes are removed with progressive tape stripping from outer to inner layers (31). Dauskardt (32) quantified the resistance of isolated SC to delamination and determined that the delamination energy increases significantly from ~3 J/m^2^ for the top SC layers to ~15 J/m^2^ for inner layers. Cryo-microtoming is also used to segment skin regions and offers depth resolved permeation data (33), but microtoming parallel to skin surface is challenging. In addition, these approaches provide only a depth profile.

Several microscopic techniques have also been used to study permeation. Confocal microscopy provides a useful means to image changes in skin structure upon permeation (34,35) but does not provide concentration information nor can it identify particular chemical entities. Confocal fluorescence imaging of an exogenous species often requires a fluorescent label for detection. The permeation properties of the exogenous agent are likely to be altered by addition of the label. In comparison, IR and Raman imaging approaches offer several advantages. The combination of molecular structure information from both the exogenous agent and the endogenous skin components offers a unique opportunity to track the spatial distribution of the effect of the exogenous material on skin barrier properties along with other potential structural alterations. In addition, confocal Raman and IR imaging provide spatial resolution of ~2 or 10 μm, respectively, somewhat worse than confocal fluorescence microscopy but without the requirement for fluorescent labels. Finally, changes in the concentrations of the exogenous species are monitored from band intensities, and as shown in Fig. 4, IR imaging can provide essentially absolute concentrations with an accuracy that is determined by the transferability of the extinction coefficients and the thickness of skin sections.

The current work presents one of the first reports delineating the spatial distribution of the concentration of a permeant, SDS, across the SC, VE and dermis. SDS-d_25_ concentrations as high as 1,000 mM in the SC compared to 40 mM in the donor compartment are observed (Fig. 4), a result consistent with partition coefficients of ~500 measured by Downing *et al.* (36) for SDS (0.1 wt%) partitioning into liposomes constructed from stratum corneum lipids. Evidently, the SC has a substantial concentrating effect on SDS-d_25_. In addition, studies of SDS depth profiles in the SC by tape stripping (13,37) report a rapid decrease in concentration with depth, also consistent with the current work (Fig. 5).

One useful application of our quantitative IR imaging data is the ability to perform a mass balance calculation. A description of our procedure and assumptions, along with a table of detailed mass balance results are provided in the Supplementary Material. Overall, our results indicate that we have detected 5–16 % of the applied dosage in porcine skin and 1–17 % in human skin, excluding the 34 °C 40 h treatment.

Comparing the current results with the investigations of Fullerton *et al.* (38), who utilized Franz diffusion cells and patch test techniques combined with radioactivity-based detection methods to track SDS permeation, is useful. While studies of permeation in deeper skin layers are scarce, Fullerton reports significant SDS levels in the dermis, with substantial variation among donors. The total amount of SDS in the epidermis was 2-30-fold greater than in the dermis following 24 h of patch application. More SDS was found in the lower regions of skin and in the recipient phase after 48 h compared to 24 h. Generally, the results are qualitatively consistent with the current work (Figs. 4 and 5 and mass balance in Supplementary Material) with the added information concerning SDS concentrations and more detailed spatial distributions now available from the IR imaging experiments.

The different temperature effects are manifest in the 24 h incubation experimental results (see Fig. 4, 3rd row) in which the human skin RT experiment displays significantly less SDS-d_25_ in the SC compared to the remaining 3 panels. This observation is consistent with prior studies from this lab and others (6,7,21,39) reporting the presence of a larger proportion of orthorhombically packed lipids in human SC at RT. In the current work, we also observed that porcine SC lipids are more disordered than human at RT as shown by the endogenous ν_sym_CH_2_ frequencies imaged in Fig. 6. In addition, the averaged ν_sym_CD_2_ frequencies in the SC displayed in Fig. 8a further suggest that porcine SC lipids are less ordered, as they display weaker ordering effects on SDS-d_25_ chains compared to human SC. Overall, it seems quite likely that the orthorhombic packing in human SC presents a stronger barrier to SDS-d_25_ permeation than the predominance of hexagonal packing present in porcine SC.

Although it would normally be of substantial interest to fully analyze the effects of SDS-d_25_ on SC lipid packing and conformational order, the technology employed in the current study limits the scope of the information available (see Materials and Methods). ν_sym_CH_2_ was the only accessible region available to track endogenous SC lipid chain structural properties. This parameter failed to provide statistically significant conclusions regarding the effects of SDS-d_25_ treatment time and temperature on SC lipid order. Several possibilities for this observation are suggested below. First, SDS may affect SC lipid structure by modifying packing order more significantly than conformational order. Second, lipid lamellar phases, whose IR spectra/structure correlations are unknown but identified by small angle x-ray diffraction (2,3), may be perturbed by SDS. Lastly, the amount of SC lipids affected by SDS may be too small to detect.

An alternative approach, using the ν_sym_CD_2_ frequencies of SDS-d_25_ to probe the SC lipid environment, is proposed. As shown in Fig. 4, SDS-d_25_ permeates beyond both porcine and human SC at 34 °C after 24 and 40 h treatment. The CD_2_ frequencies for these two samples in the SC region are considerately higher than their counterparts at room temperature (Fig. 8a). This shift in SDS-d_25_ conformational order may be related to the damaging effects of SDS-d_25_ on the skin barrier, especially on the SC lipid organization. Coupled with the lack of a major SDS-induced perturbation of keratin structure (Fig. 7), an intercellular pathway is tentatively suggested. At short times or (relatively) low temperature, lower amounts of SDS-d_25_ permeate into skin and its acyl chain order increases due to mixing with the ordered SC lipids. As mentioned earlier, at relatively higher temperature (34 °C) SDS-d_25_ permeation may be enhanced by the different state of the endogenous lipid packing in the SC. The increased amount of SDS-d_25_ in the SC combined with faster kinetics of skin/SDS-d_25_ interaction at higher temperature lead to more severe disruption of the endogenous SC lipid acyl chain conformational order and/or packing. As a result, the ordering effect of SC on SDS-d_25_ acyl chains diminishes. This is consistent with our parallel study (21) of isolated SC by transmission IR, which demonstrated that the most significant effects of SDS-d_25_ incorporation were the removal and/or disordering of SC lipids packed in orthorhombic phases and the lowering of the orthorhombic to hexagonal phase transition temperature.

The mode of surfactant permeation has long been debated. It had been generally accepted that SDS permeates as a monomer. Polymers and milder co-surfactants have been added to SDS to decrease the irritation associated with a pure SDS system (40–42) by reducing the SDS monomer concentration. Other studies have suggested that the monomer penetration model doesn’t completely explain how SDS damages skin. Moore *et al*. (43) reported that SDS increased transepidermal current at concentrations above the CMC. James-Smith *et al.* (44) found that skin conductivity and permeated SDS concentration in skin correlated with the concentration of monomers and sub-micellar aggregates. The current IR imaging data offer some insights into this issue. As noted above, the methylene stretching frequency of SDS-d_25_ is sensitive to chain conformational ordering. At temperatures above the Krafft point (~18 °C), the SDS-d_25_ chains in micellar solutions are fairly disordered with a ν_sym_CD_2_ frequency of ~2,096 cm^−1^. As shown in Fig. 8a, SDS-d_25_ methylene stretching frequencies are several wavenumbers lower in the SC compared to solution phase, indicating that the SDS-d_25_ residing in the SC exists in a more ordered state than in micelles. Within the dermis, however, the range of ν_sym_CD_2_ frequencies (Fig. 8b) is similar to that observed for the micellar solutions. With the average SDS-d_25_ dermal concentration (~36.9 mM) greater than the CMC, we cannot rule out the reforming of micelles in the dermis, although this does seem unlikely.

## CONCLUSION

This study takes the advantage of confocal Raman microscopy and IR imaging to image and quantify the spatial distribution of SDS in skin. SDS distributes heterogeneously among skin regions and within each skin region. The preferential partitioning of SDS into skin resulted in an SDS concentration in the SC higher than the donor solution. Substantial SDS levels are also found in the dermal region after a prolonged incubation time. The availability of a complete vibrational spectrum at each sampling point of an IR and confocal Raman image offers unique molecular level information concerning the changes in physical properties of SDS and its damaging effects on the skin barrier. Careful examination of these data also provides valuable insights into the surfactant permeation mode and delivery pathway. This study suggests SDS exists in a more ordered state in the SC compared to its micellar form and offers evidence to support an intercellular lipid permeation pathway for SDS in the SC.

## Electronic supplementary material

Below is the link to the electronic supplementary material.

ESM 1 (DOC 108 kb)

## ABBREVIATIONS

CMC: critical micelle concentration
Der: dermis
SC: stratum corneum
SDS: sodium dodecyl sulfate
SDS-d_25_: acyl chain perdeuterated SDS
VE: viable epidermis
ν_asym_CH_2_: asymmetric methylene stretching mode
ν_sym_CH_2_: symmetric methylene stretching mode
