# Supplementary material for: Imaging the Distribution of Sodium Dodecyl Sulfate in Skin by Confocal Raman and Infrared Microspectroscopy
Source: Pharm Res. 2012 Apr 4;29(8):2189–201. doi: 10.1007/s11095-012-0748-y (PMC3399083; doi:10.1007/s11095-012-0748-y)
Supplement: Supplementary file 1 — (DOC 108 kb) [file 11095_2012_748_MOESM1_ESM.doc]

**Supplementary: Mass balance of SDS-d_25_ in different skin regions**

*Assumptions and Procedures*

It is assumed that the SDS-d_25_ concentration determined by IR imaging from a small sample area (with a 200μm*7μm area parallel to skin surface and 300μm depth into skin) is representative of the SDS-d_25_ distribution in the entire skin sample (a cylindrical skin sample with ~8mm diameter and ~2.5mm in depth). The detection limit for SDS-d_25_ in skin under our conditions is 17 mM.

Different skin regions (SC vs VE vs dermis) are delineated by factor analysis based on the νCH stretching band region between 2830-3000 cm^-1^. The average depth of SDS-d_25_ permeation in each skin region is calculated from the number of pixels with detectable ν_sym_CD_2_ (peak height at 2098 cm^-1^>1.9 milliabsorbance) signal in each region. For the human skin sample treated at 34°C for 40h, SDS-d_25_ was observed to permeate through the entire dermal region currently sampled with a concentration of approximately 32 mM. The thickness of the dermis for this sample is used as the depth of permeation in its dermal region and is calculated by subtracting the depth of SC and VE from the thickness of the entire skin sample of 2.5 mm. The average SDS-d_25_ concentration for each skin region with detectable ν_sym_CD_2_ is calculated from the ν_sym_CD_2_ integrated peak area as detailed in the experimental section.

The amount of SDS-d_25_ in each skin region is calculated as follows:

Total amount of SDS-d_25_ in each skin region=average SDS-d_25_ concentration in each skin region* average depth of SDS-d_25_ permeation in this region* surface area of the treated sample * molecular weight of SDS-d_25_, where the surface area of the treated sample=π*(diameter of donor chamber/2)^2^=π*(0.8cm/2)^2^=50.24 cm^2^. The molecular weight of SDS-d_25_=313.38 g/mol. The residual amount of SDS-d_25_ in the donor chamber was not quantified.

The total amount applied: 12.5 mg/mL*0.12 mL=1.5 mg. The % of applied dose=total amount of SDS-d_25_ in each skin region/total amount applied.

*Results*

***Table S-1 Mass balance of SDS-d_25_ in different porcine skin regions.***

|  | Treatment | 24C 3h | 24C 24h | 24C 40h | 34°C3h | 34°C 24h | 34°C 40h |
| --- | --- | --- | --- | --- | --- | --- | --- |
| SC | Ave conc, mM | 246.3 | 229.2 | 146.3 | 261.4 | 333.8 | 183.8 |
|  | Ave thickness, um | 19.5 | 24.8 | 38.9 | 18.0 | 40.0 | 37.9 |
|  | Total amount | 0.0756 | 0.0895 | 0.0896 | 0.0741 | 0.2102 | 0.1097 |
|  | % of applied dose | 5.0% | 6.0% | 6.0% | 4.9% | 14.0% | 7.3% |
| VE | Ave conc, mM | 0 | 0 | 0 | 0 | 48.0 | 55.5 |
|  | Ave thickness with SDS, um | 0 | 0 | 0 | 0 | 30.7 | 63.5 |
|  | Total amount | 0 | 0 | 0 | 0 | 0.023 | 0.055 |
|  | % of applied dose | 0 | 0 | 0 | 0 | 1.5% | 3.7% |
| Dermis | Ave conc, mM | 0 | 0 | 0 | 0 | 0 | 36.9 |
|  | Ave thickness with SDS, um | 0 | 0 | 0 | 0 | 0 | 62.9 |
|  | Total amount | 0 | 0 | 0 | 0 | 0 | 0.037 |
|  | % of applied dose | 0 | 0 | 0 | 0 | 0 | 2.4% |
| Skin | Total amount | 0.0756 | 0.0895 | 0.0896 | 0.0741 | 0.2332 | 0.2087 |
|  | % of applied dose | 5.0% | 6.0% | 6.0% | 4.9% | 15.5% | 13.4% |

**Table S-2 Mass balance of SDS-d_25_ in different human skin regions.**

|  | Treatment | 24C 3h | 24C 24h | 24C 40h | 34°C3h | 34°C 24h | 34°C 40h |
| --- | --- | --- | --- | --- | --- | --- | --- |
| SC | Ave conc, mM | 103.5 | 83.32 | 368.7 | 113.9 | 674.6 | 673.4 |
|  | Ave thickness, um | 11.9 | 11.5 | 17.6 | 15.6 | 18.0 | 19.1 |
|  | Total amount | 0.0194 | 0.0151 | 0.1022 | 0.0280 | 0.01911 | 0.2025 |
|  | % of applied dose | 1.3% | 1.0% | 6.8% | 1.9% | 12.7% | 13.5% |
| VE | Ave conc, mM | 0 | 0 | 53.0 | 0 | 86.9 | 216.2 |
|  | Ave thickness with SDS, um | 0 | 0 | 23.4 | 0 | 43.6 | 56.1 |
|  | Total amount | 0 | 0 | 0.0195 | 0 | 0.0597 | 0.1910 |
|  | % of applied dose | 0 | 0 | 1.3% | 0 | 4.0% | 12.73% |
| Dermis | Ave conc, mM | 0 | 0 | 0 | 0 | 0 | 32.0 |
|  | Ave thickness, um | 0 | 0 | 0 | 0 | 0 | 2425 |
|  | Total amount | 0 | 0 | 0 | 0 | 0 | 1.2218 |
|  | % of applied dose | 0 | 0 | 0 | 0 | 0 | 81.5% |
| Skin | Total amount | 0.0194 | 0.0151 | 0.01217 | 0.028 | 0.07881 | 1.6153 |
|  | % of applied dose | 1.3% | 1.0% | 8.1% | 1.9% | 16.7% | 107.6% |
